# The Yin and Yang of SagS: Distinct Residues in the HmsP Domain of SagS Independently Regulate Biofilm Formation and Biofilm Drug Tolerance

**DOI:** 10.1128/mSphere.00192-18

**Published:** 2018-05-30

**Authors:** Jozef Dingemans, Bandita Poudyal, Holger Sondermann, Karin Sauer

**Affiliations:** a Department of Biological Sciences, Binghamton Biofilm Research Center, Binghamton University, Binghamton, New York, USA; b Department of Molecular Medicine, College of Veterinary Medicine, Cornell University, Ithaca, New York, USA; Carnegie Mellon University

**Keywords:** BfiS, BrlR, Rosetta modeling, biofilm drug tolerance, biofilm formation

## Abstract

The formation of inherently drug-tolerant biofilms by the opportunistic pathogen Pseudomonas aeruginosa requires the sensor-regulator hybrid SagS, with Δ*sagS* biofilms being unstructured and exhibiting increased antimicrobial susceptibility. Recent findings indicated SagS to function as a switch to control biofilm formation and drug tolerance independently. Moreover, findings suggested the periplasmic sensory HmsP domain of SagS is likely to be the control point in the regulation of biofilm formation and biofilm cells transitioning to a drug-tolerant state. We thus asked whether specific amino acid residues present in the HmsP domain contribute to the switch function of SagS. HmsP domain residues were therefore subjected to alanine replacement mutagenesis to identify substitutions that block the sensory function(s) of SagS, which is apparent by attached cells being unable to develop mature biofilms and/or prevent transition to an antimicrobial-resistant state. Mutant analyses revealed 32 residues that only contribute to blocking one sensory function. Moreover, amino acid residues affecting attachment and subsequent biofilm formation but not biofilm tolerance also impaired histidine kinase signaling via BfiS. In contrast, residues affecting biofilm drug tolerance but not attachment and subsequent biofilm formation negatively impacted BrlR transcription factor levels. Structure prediction suggested the two sets of residues affecting sensory functions are located in distinct areas that were previously described as being involved in ligand binding interactions. Taken together, these studies identify the molecular basis for the dual regulatory function of SagS.

**IMPORTANCE** The membrane-bound sensory protein SagS plays a pivotal role in P. aeruginosa biofilm formation and biofilm cells gaining their heightened resistance to antimicrobial agents, with SagS being the control point at which both pathways diverge. Here, we demonstrate for the first time that the two distinct pathways leading to biofilm formation and biofilm drug tolerance are under the control of two sets of amino acid residues located within the HmsP sensory domain of SagS. The respective amino acids are likely part of ligand binding interaction sites. Thus, our findings have the potential not only to enable the manipulation of SagS function but also to enable research of biofilm drug tolerance in a manner independent of biofilm formation (and vice versa). Moreover, the manipulation of SagS function represents a promising target/avenue open for biofilm control.

## Abstract (precis)

The membrane-bound sensory protein SagS plays a pivotal role in P. aeruginosa biofilm formation and biofilm cells gaining their heightened resistance to antimicrobial agents, with SagS being the control point at which both pathways diverge. Here, we demonstrate for the first time that the two distinct pathways leading to biofilm formation and biofilm drug tolerance are under the control of two sets of amino acid residues located within the HmsP sensory domain of SagS. The respective amino acids are likely part of ligand binding interaction sites. Thus, our findings have the potential not only to enable the manipulation of SagS function but also to enable research of biofilm drug tolerance in a manner independent of biofilm formation (and vice versa). Moreover, the manipulation of SagS function represents a promising target/avenue open for biofilm control.

## INTRODUCTION

Biofilms are composed of microorganisms attached to a solid surface and encased in an exopolysaccharide (EPS) matrix of their own synthesis (1–3). These matrix-enclosed aggregates are associated with more than >60% of all recalcitrant and chronic infections. Infections caused by bacterial biofilms have been associated with a number of medical conditions, including periodontal disease, endocarditis, osteomyelitis, cystic fibrosis, and indwelling device-related biofilm infections (4). Bacteria living in biofilms can be up to 1,000 times more tolerant to antibacterial compounds than their planktonic counterparts (5–7). It is thus not surprising that conventional therapies have proven inadequate in the treatment of many (if not most) chronic biofilm infections due to the extraordinary resistance of biofilms to antimicrobial treatments (4, 8, 9). Biofilm antimicrobial resistance is distinct from commonly known mechanisms such as plasmid-borne resistance markers or resistance conferred by mutation (10–14), indicating that the mechanisms involved in the recalcitrance of biofilms against antimicrobials may differ from the mechanisms responsible for antimicrobial resistance in planktonic bacteria. This recalcitrance of biofilm cells to killing by antimicrobial agents has been termed “tolerance” (8, 9). Although several mechanisms have been postulated to explain reduced susceptibility to antimicrobials in bacterial biofilms, the current notion is that biofilm drug tolerance is multifactorial as only a combination of different mechanisms could account for the level of resilience against antimicrobial agents observed in biofilm communities. These factors include reduced metabolic and divisional rates (15–18), starvation-induced growth arrest (19), the presence of persister cells that neither grow nor die in the presence of microbicidal antibiotics (9, 20–23), and restricted penetration of antimicrobials into a biofilm (12, 14, 24–29).

**FIG 1:**
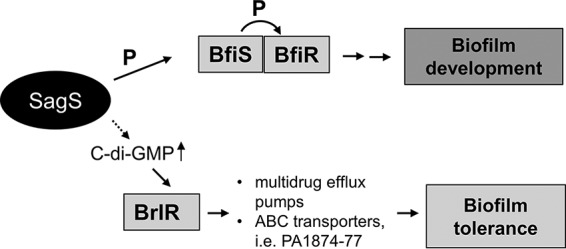
Overview of the contribution of the two-component hybrid SagS to the motile-sessile and susceptible-resistance switches by P. aeruginosa cells. Upon P. aeruginosa transition to surface-associated growth, SagS directly interacts with and phosphorylates the TCS BfiSR, thus enabling surface-associated cells to transition to the irreversible attachment stage (31, 36). Moreover, transition to the irreversible attachment stage, regulated by SagS, marks the timing when surface-associated cells gain their heightened resistance to antimicrobial agents, with inactivation of *sagS* having been previously demonstrated to correlate with biofilm cells but not planktonic cells being more susceptible to antimicrobial agents (30, 36). SagS contributes to the activation of biofilm tolerance via c-di-GMP and the transcriptional regulator BrlR.

Recent findings furthermore, indicate that biofilms themselves are not simply a diffusion barrier to these antibiotics, but rather the bacteria within these microbial communities employ distinct mechanisms to resist the action of antimicrobial agents. This is supported by findings in Pseudomonas aeruginosa suggesting drug tolerance to be a function of the progression of biofilm development. We previously demonstrated that in P. aeruginosa, at least two proteins are required for drug tolerance: the two-component system (TCS) hybrid SagS and the c-di-GMP-responsive transcriptional regulator BrlR. SagS enables the transition from the free-floating, planktonic to sessile biofilm mode of growth and the switch from an antimicrobial-susceptible to highly tolerant state (6, 7, 30–33). More specifically, SagS contributes to biofilm formation via hierarchical phosphotransfer-based signaling between SagS and the TCS BfiSR (31, 34–36) and biofilm tolerance by activating the biofilm-specific transcriptional regulator BrlR in a manner independent of phosphotransfer, biomass accumulation, biofilm architecture, and the later stages of biofilm maturation (30, 36, 37) (Fig. 1). BrlR, in turn, activates the expression of several multidrug efflux pumps and ABC transporters (6, 7, 32, 38), with the ABC transport system PA1874-77 directly contributing to the drug tolerance of biofilms (38) (Fig. 1). Taken together, our findings indicate SagS acts as a switch to control biofilm formation and drug tolerance. However, little is known about how this is accomplished by SagS.

**FIG 2:**
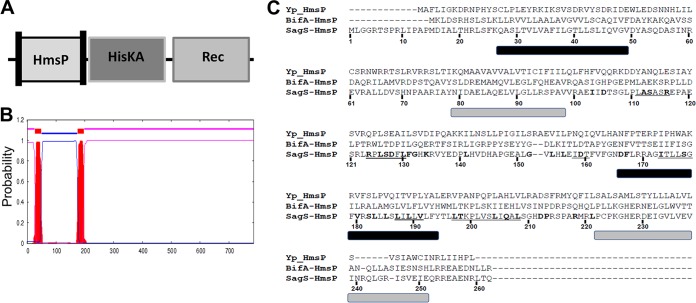
SagS harbors a sensory HmsP domain flanked by two membrane-spanning regions that are similar to the HmsP domains of BifA and HmsP. (A) Overview of SagS domains. Vertical bars within the HmsP domain indicate transmembrane helices. (B) Localization of transmembrane helices present in SagS as determined using the TMHMM software for prediction of transmembrane helices in proteins (http://www.cbs.dtu.dk/services/TMHMM-2.0/). (C) Alignment of the sensory HmsP domain of SagS with the HmsP domains of BifA from P. aeruginosa and HmsP from Y. pestis. Residues subjected to site-directed mutagenesis are highlighted in boldface; cassette mutations are underlined. Predicted membrane-spanning regions are shown by bars below the alignments: black bars are based on TMHMM and PHYRE2, and gray bars are based on secondary structure prediction of HmsP by Y. pestis.

SagS harbors three domains: an N-terminal HmsP domain, a histidine kinase (HisKA) domain, and a C-terminal response regulator receiver (Rec) domain (Fig. 2A). The HmsP domain of SagS, flanked by two transmembrane helices (Fig. 2B), is homologous to the N-terminal domains of the biofilm formation regulators BifA and HmsP in P. aeruginosa and Yersinia pestis, respectively (39–41), and represents a sensory domain. Using SagS domain constructs, we previously demonstrated that the two-component hybrid SagS enables biofilm formation and recalcitrance of biofilm cells to antimicrobial agents via two distinct regulatory circuits (36). Moreover, we demonstrated that the periplasmic sensory domain HmsP of SagS contributed to the switch to control biofilm formation and drug tolerance (36). Given that SagS is a membrane-bound two-component hybrid, with computational analyses suggesting the N-terminal sensory HmsP domain of SagS to extend into the periplasmic space to perceive specific signals or cues, the findings suggested the periplasmic HmsP domain is a control point in the regulation of biofilm formation and biofilm cells transitioning to a drug-tolerant state.

We therefore set out to identify amino acid residues in the HmsP domain that specifically contribute to the switch function of SagS and to determine whether these mutations would contribute to one or both signaling mechanisms. We surmised that if such amino acid residues exist, substitution for such amino acids may block the sensory function(s) of HmsP, apparent by attached cells being unable to (i) develop mature biofilms and/or (ii) prevent transition to an antimicrobial-resistant state.

## RESULTS

### Selection of amino acid residues of the HmsP domain of SagS for mutational analysis.

The HmsP domain has not been subjected to structure-function analysis. To identify amino acid residues in the HmsP domain contributing to SagS function(s), we took advantage of the homology between the SagS-HmsP domain and the N-terminally located HmsP domains of the biofilm formation regulators BifA and HmsP in P. aeruginosa and Yersinia pestis, respectively (Fig. 2C). Given that all three proteins have sensory functions (39–41), we reasoned that residues that are similar or identical between all HmsP domains may contribute to their sensory function. We furthermore, focused on amino acid residues predicted to be located between the two membrane-spanning regions (Fig. 2B and C), as these regions are predicted to be located in the periplasmic space (Fig. 2C) and thus, anticipated to perceive specific signals or cues. Using these criteria, we selected a total of 6 amino acid residues that were identical in all HmsP domains, including the Y. pestis HmsP domain, and 32 amino acid residues that were identical or similar in at least two HmsP domains. Additionally, the alignment indicated 6 regions located at or between the two membrane-spanning regions at which 2 or more residues in a row were conserved (underlined in Fig. 2C). The selected 38 single amino acid residues and 6 extended motifs were subjected to alanine substitution (with natural alanine residues being substituted for with serine). We thus, generated a total of 44 *sagS* variants (referred to as *sagS*_*mut*_), which were subsequently cloned into pMJT-1 and transferred into the Δ*sagS* mutant strain.

### Select SagS_mut_ variants fail to restore the attachment phenotype of Δ*sagS* mutant strain to wild-type levels.

Given that in Pseudomonas aeruginosa the TCS hybrid SagS regulates both (i) the transition to biofilm formation and (ii) the transition to cells gaining their enhanced tolerance to antimicrobial agents, we reasoned that substitution of amino acids that contribute to SagS function would impair the switch function, which is apparent by attached cells being unable to (i) develop mature biofilms and (ii) transition to a resistant state.

**FIG 3:**
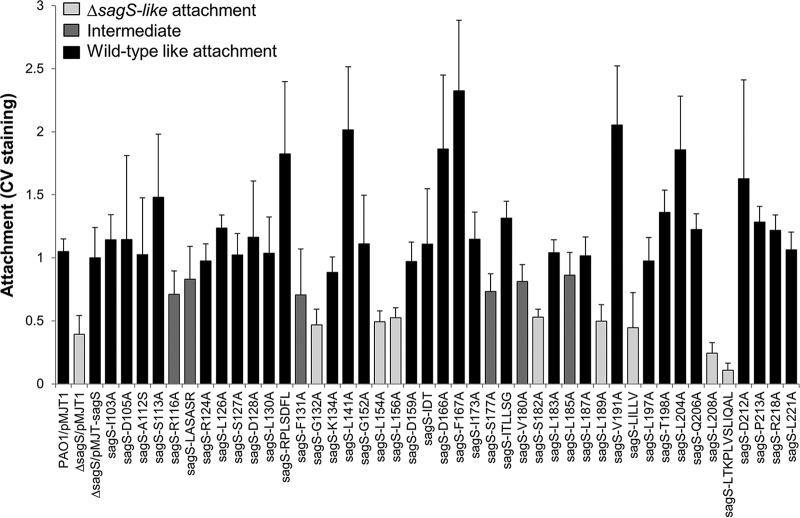
Attachment capabilities of Δ*sagS* mutant strains expressing *sagS* or *sagS*_*mut*_. Evaluation of attachment to a polystyrene surface by crystal violet staining following 24 h of growth under shaking conditions. Attachment is expressed relative to the Δ*sagS*/pMJT-*sagS* mutant. PAO1 and the Δ*sagS*/pMJT-1 mutant were used as controls. All assays were repeated at least three times, with each repeat consisting of 8 technical replicates. Error bars denote standard deviation. Attachment capabilities are ranked as Δ*sagS* like (significantly different from PAO1; *P* < 0.01), wild-type like, or intermediate (significantly different from PAO1 and Δ*sagS*, with relative attachment ranging from 0.7 to 0.8; *P* < 0.01).

We therefore first screened Δ*sagS* mutant strains expressing all 44 SagS variants for attachment. The screen is based on staining biofilms grown in 96-well microtiter plates for 24 h using crystal violet (CV) (42, 43), thus enabling quantitation of attachment capabilities based on biofilm biomass accumulation (Fig. 3). The Δ*sagS*/pMJT-1 and Δ*sagS*/pMJT-*sagS* mutants (31) and wild-type PAO1 harboring an empty plasmid (PAO1/pMJT1) were used as controls. Of the 44 Δ*sagS/*pMJT-*sagS*_*mut*_ strains tested, 32 restored attachment by the Δ*sagS* mutant strain to wild-type levels following 24 h of attachment. In contrast, single alanine substitution at amino acid residue positions F131, G132, L154, L156, S182, L189, and L208 and substitutions at two short sequence motifs, LILLV and LTKPLVSLIQAL, coincided with significantly reduced surface adherence, with CV staining being comparable to that noted for the Δ*sagS* mutant harboring an empty plasmid. Moreover, substitutions of R116, S117, V180, L185, and the LASASR motif coincided with the Δ*sagS*/pMJT-*sagS*_*mut*_ mutant displaying an attachment phenotype intermediary to the Δ*sagS* and Δ*sagS*/pMJT-*sagS* mutants (Fig. 3). Our findings suggest that out of 44 SagS variants tested, 14 variants either failed to restore or only partly restored attachment of Δ*sagS* mutant strains to wild-type levels (Table 1).

**TABLE 1:** Phenotypic characterization of SagS variants^a^

Substitution	Restoration of Δ*sagS* biofilm-related phenotypes to wild-type levels	
	Attachment	Susceptibility to tobramycin
I103	Yes	Yes
D105	Yes	No
A112	Yes	No
S113	Yes	Yes
R116	Intermediate	Yes
R124	Yes	Yes
L126	Yes	No
S127	Yes	No
D128	Yes	No
L130	Yes	Yes
F131	No	Yes
G132^b^	No	No
K134	Yes	Yes
L141	Yes	Intermediate
G152	Yes	Intermediate
L154	No	Yes
L156^b^	No	No
D159	Yes	No
D166	Yes	No
F167	Yes	Intermediate
I173	Yes	Intermediate
S177	Yes	Yes
V180	Intermediate	Yes
S182	No	Yes
L183	Yes	No
L185	Intermediate	Yes
L187	Yes	No
L189^b^	No	Intermediate
V191	Yes	Yes
L197	Yes	Intermediate
T198	Yes	Yes
L204	Yes	No
Q206	Yes	No
L208	No	Yes
D212	Yes	Yes
P213	Yes	No
R218	Yes	Yes
L221	Yes	No
R218	Yes	Intermediate
LILLV	No	Yes
LTKPLVSLIQAL^b^	No	No
IDT	Yes	No
LASASR	Intermediate	Yes
RPLSDFL	Yes	No
ITLLSG	Yes	Intermediate

a Phenotypic characterization was based on attachment capabilities and susceptibility of biofilms to tobramycin. Wild-type and Δ*sagS* mutant strains were used as controls. SagS variants with substitutions with underlined residues were chosen for further analyses (see Fig. 4, 5, and 7). Attachment phenotypes correspond to data shown in Fig. 3, while the susceptibility phenotypes are based on data shown in Fig. 6. “Yes” indicates no significant difference from PAO1, “No” indicates significantly different from PAO1, and “Intermediate” indicates significantly different, but values were in between those obtained for PAO1 or the Δ*sagS*/pMJT-*sagS* and *ΔsagS* mutants.

b These SagS variants failed to restore both attachment and susceptibility phenotypes by Δ*sagS* mutant biofilms to wild-type levels, so the respective variants were excluded from subsequent analyses.

### SagS variants that fail to restore attachment likewise fail to restore the biofilm architecture of the *ΔsagS* mutant to wild-type levels.

We next asked whether select amino acid residues likewise contribute to biofilm development. Based on the attachment phenotype, we surmised a role of amino acid residues F131, G132, L154, L156, S182, L189, and L208 (as well as LILLV) in biofilm development. To further explore the contribution of these residues in biofilm formation, we selected SagS_F131A and SagS_L154A, which failed to restore attachment of Δ*sagS* mutant strains to the wild-type level. In addition, we selected SagS_mut_ variants found to either partly or fully restore attachment by Δ*sagS* mutant strains to wild-type levels. Specifically, we selected SagS_mut_ variants harboring substitutions in R116, D105, L126, and L141. To confirm the production of these 6 SagS_mut_ variants, protein levels were analyzed by immunoblot analysis using cell-free total extract (Fig. 4A). With the exception of SagS_F131A, which was consistently found to be reduced by approximately 50% relative to SagS, all other variants were produced at levels comparable to wild-type SagS (Fig. 4A).

**FIG 4:**
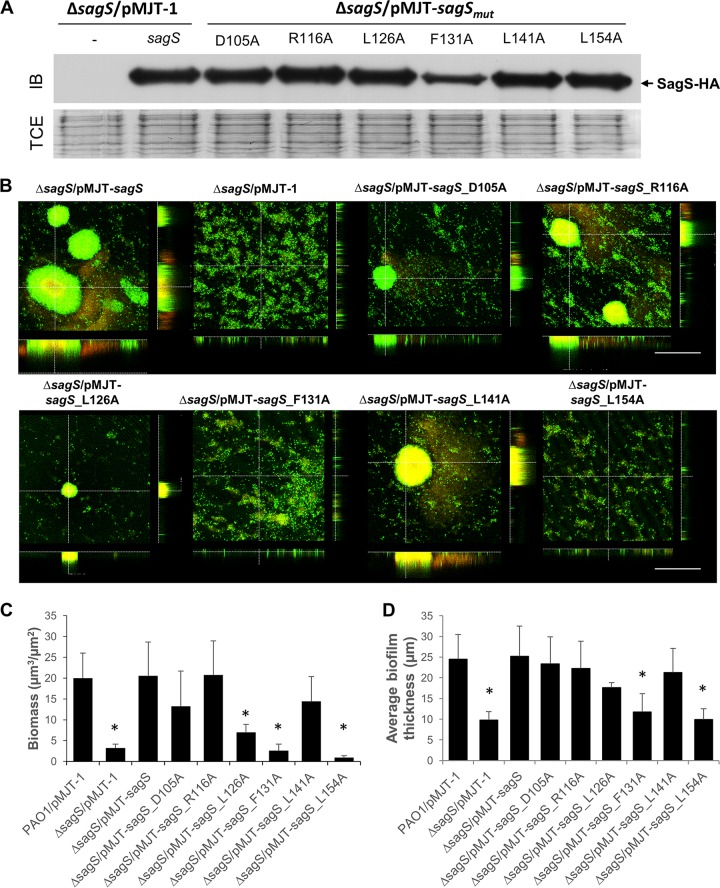
Biofilm architecture of **Δ***sagS* mutant strains expressing *sagS* or select *sagS*_*mut*_ variants. (A) Representative image of an immunoblot (IB) demonstrating the presence of SagS in total cell extracts (TCEs) obtained from Δ*sagS* mutant strains overexpressing *sagS* or *sagS*_*mut*_ grown planktonically to the exponential phase. A Δ*sagS* mutant strain harboring the empty plasmid pMJT-1 was used as a control. A total of 15 µg total cell extract was loaded. Immunoblots were probed for the presence of SagS using antihemagglutinin (anti-HA) antibodies. The corresponding SDS-PAGE gel image (TCE) obtained posttransfer demonstrates equal loading. (B) Representative confocal images showing the architecture of biofilms formed by P. aeruginosa Δ*sagS* and Δ*sagS* mutant strains overexpressing *sagS* or *sagS*_*mut*_. Biofilms were grown for 3 days in 5-fold-diluted LB medium, after which time confocal images were acquired. Biofilms were stained with the LIVE/DEAD BacLight viability stain (Life Technologies, Inc.). White bars = 100 µm. (C and D) Biofilm biomass (C) and biofilm height (D) of Δ*sagS* mutant strains overexpressing *sagS* and *sagS*_*mut*_ grown for 3 days, as determined using confocal images and subsequent COMSTAT analysis. Biofilms by PAO1 and the Δ*sagS* mutant harboring the empty vector pMJT-1 were used as controls. All assays were repeated at least three times, with a minimum of 6 images being acquired. Error bars indicate standard deviation. *, significantly different from the values for the P. aeruginosa Δ*sagS*/pMJT-*sagS* mutant (*P* ≤ 0.01).

Considering that expression of *sagS*_D105A, -L126A, and -L141A restored attachment while expression of *sagS*_F131A, -L154A, and -R116A coincided with significantly reduced attachment relative to intact *sagS*, we anticipated only the expression of *sagS*_*mut*_ variants *sagS*_D105, -L126, and -L141 to restore biofilm formation by Δ*sagS* biofilms to wild-type levels. Biofilms were grown in 24-well microtiter plates for 3 days, with the medium being replaced every 12 h. The Δ*sagS*/pMJT-1 and Δ*sagS*/pMJT-*sagS* strains (31) and PAO1 harboring an empty plasmid (PAO1/pMJT1) were used as controls. Under the conditions tested and in agreement with previous findings (31, 34), the Δ*sagS* mutant formed thin biofilms lacking large cellular aggregates and harboring >5-fold-less biomass than biofilms formed by the isogenic parental strain, while overexpression of intact *sagS* restored the biofilm architecture by Δ*sagS* to wild-type levels (Fig. 4B to D; see Fig. S1 in the supplemental material). Likewise, expression of *sagS*_D105, -L126, and -L141 coincided with biofilms characterized by large cellular aggregates that are comparable in average thickness and maximum height to wild-type and complemented Δ*sagS*/pMJT-*sagS* mutant biofilms (Fig. 4B to D and Fig. S1). Surprisingly, expression of the *sagS*_R116 likewise restored biofilm formation to wild-type levels. In contrast, biofilms formed by the Δ*sagS*/pMJT-*sagS*_F131 and Δ*sagS*/pMJT-*sagS*_L154 mutants were comparable to Δ*sagS* biofilms, in that the mutant biofilms were thin, lacked large cellular aggregates, and harbored >5-fold-less biomass than biofilms formed by the isogenic parental strain (Fig. 4B to D and Fig. S1).

10.1128/mSphere.00192-18.1 FIG S1  Biofilm maximum thickness. Shown is the biofilm maximum thickness of Δ*sagS* mutant strains overexpressing *sagS* and *sagS*_*mut*_ grown for 3 days, as determined using confocal images and subsequent COMSTAT analysis. Biofilms by the PAO1 wild type and Δ*sagS* mutant harboring the empty vector pMJT-1 were used as controls. All assays were repeated at least three times, with a minimum of 6 images being acquired. Error bars indicate standard deviation. *, significantly different from the values for the P. aeruginosa Δ*sagS*/pMJT-*sagS* mutant (*P* ≤ 0.01). Download FIG S1, PDF file, 0.1 MB. Copyright © 2018 Dingemans et al. 2018 Dingemans et al. This content is distributed under the terms of the Creative Commons Attribution 4.0 International license.

### Substitutions for F131 and L154 with alanine coincide with impaired phosphorylation of BfiS.

**FIG 5:**
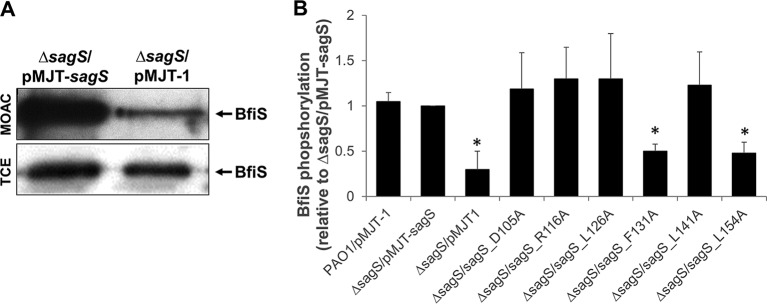
SagS variants contributing to attachment and biofilm formation also contribute to BfiS phosphorylation. (A) Detection of BfiS in total cell extracts (TCEs) and in MOAC-enriched phosphoproteomes (MOAC) of P. aeruginosa Δ*sagS* cells harboring the empty vector pMJT-1 or overexpressing *sagS*. Cell-free total cell extracts were obtained from Δ*sagS*/pMJT-1 and Δ*sagS*/pMJT*-sagS* cells grown as biofilms. For MOAC samples, the entire MOAC eluate concentrated using methanol-chloroform precipitation was loaded. A representative image is shown. (B) Relative level of BfiS phosphorylation, based on relative intensity of protein bands detectable following probing for BfiS with anti-V5 antibodies and subsequent analysis using ImageJ (56). Experiments were carried out in triplicate. Error bars denote standard deviation. *, significantly different from the values for the Δ*sagS*/pMJT*-sagS* mutant (*P* ≤ 0.01), as determined by ANOVA and Prism5.

The interaction and phosphotransfer between SagS and BfiS have been implicated in the regulation of biofilm formation (31, 34, 35). To determine whether restoration of biofilm formation via expression of *sagS*_*mut*_ variants is dependent on the phosphotransfer between SagS and BfiS, we determined the phosphorylation status of BfiS using metal oxide affinity chromatography (MOAC). Under the conditions tested and in agreement with previous findings (31), little to no BfiS phosphorylation was noted in Δ*sagS* biofilm cells, while phosphorylated BfiS was detectable in Δ*sagS* biofilm cells expressing full-length *sagS* (Fig. 5A). Likewise, expression of *sagS*_D105A, -R116A, -L126A, and -L141A in the Δ*sagS* mutant restored BfiS phosphorylation in Δ*sagS* biofilm cells, in a manner similar to overexpression of *sagS* (Fig. 5B). In contrast, significantly reduced levels of phosphorylated BfiS were detected in Δ*sagS* cells expressing *sagS*_F131A and *sagS*_L154A, with BfiS phosphorylation levels being comparable to those detected in in Δ*sagS* biofilm cells (Fig. 5B). Our findings suggest that biofilm development by P. aeruginosa coincides with phosphotransfer-based signaling between SagS and the TCS BfiSR and that substitution of residues affecting attachment and biofilm formation interferes with BfiS phosphorylation.

### Identification of SagS amino acid residues that contribute to P. aeruginosa biofilms gaining their heightened resistance to antimicrobial agents.

As indicated above, SagS contributes to both (i) the transition to biofilm formation and (ii) the transition to cells gaining their enhanced tolerance to antimicrobial agents. Given that 14 substitutions negatively affected attachment (Fig. 3), with two furthermore confirmed to affect biofilm formation and BfiS phosphorylation (Fig. 4 and 5), we next asked whether the same or additional amino acids likewise contributed to biofilms gaining their heightened resistance to antimicrobial agents.

**FIG 6:**
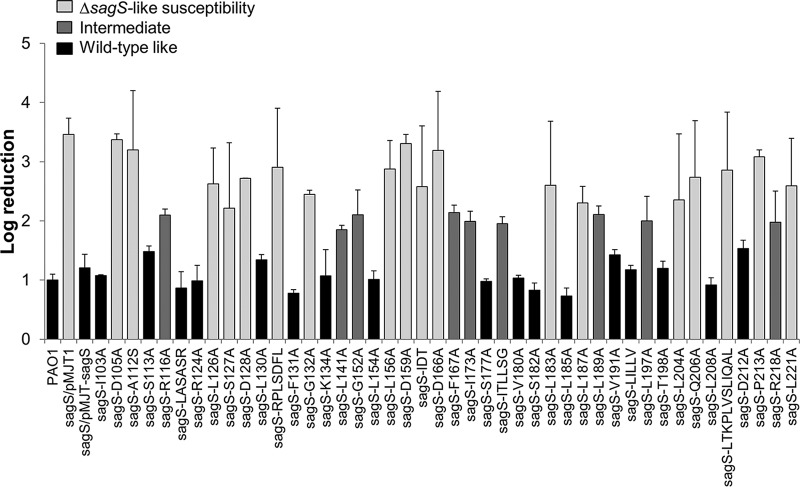
Susceptibility of Δ*sagS* mutant biofilm cells (2 days old) expressing *sagS* or *sagS*_*mut*_ to tobramycin. Biofilms by the P. aeruginosa PAO1 wild type and Δ*sagS* mutant harboring the empty plasmid pMJT-1 were used as a control. Viability was determined via CFU counts. Susceptibility is expressed as log_10_ reduction. Experiments were carried out in triplicate. Error bars denote standard deviation. Biofilm susceptibility is ranked as Δ*sagS* like (significantly different from PAO1; *P* < 0.01), wild-type like, or intermediate (significantly different from PAO1 and the Δ*sagS* mutant, with log reductions ranging from 1.8 to 2.2; *P* < 0.05).

We therefore subjected all 44 Δ*sagS/*pMJT-*sagS*_*mut*_ strains to biofilm antibiotic susceptibility assays, using 3-day-old biofilms grown under continuous flow conditions in tube reactors. Following exposure to tobramycin for 1 h, biofilms were harvested and viability of treated and untreated biofilms was determined using the CFU count. The Δ*sagS*/pMJT-1 and Δ*sagS*/pMJT-*sagS* strains (31) and PAO1 harboring an empty plasmid (PAO1/pMJT1) were used as controls. Expression of the majority of *sagS*_*mut*_ restored the susceptibility phenotype of Δ*sagS* mutant biofilms to wild-type levels, which was apparent by exposure to tobramycin only coinciding with a 1-log_10_ reduction in the viability of the respective Δ*sagS*/pMJT-*sagS*_*mut*_ biofilms (Fig. 6). A similar log_10_ reduction was noted for wild-type and Δ*sagS*/pMJT-*sagS* mutant biofilms. However, expression of *sagS*_*mut*_ variants encoding SagS harboring single substitutions at residues D105, A112, L126, S127, D128, G132, L156, D159, D166, L183, L187, L204, Q206, P213, or L221 was as susceptible to tobramycin as Δ*sagS* mutant biofilms (Fig. 6). Similar to Δ*sagS* mutant biofilms, exposure of the respective Δ*sagS*/pMJT-*sagS*_*mut*_ biofilms to tobramycin coincided with an ~3- to 3.5-log_10_ reduction. Moreover, expression of *sagS*_*mut*_ harboring the mutations in sequence motifs IDT, RPLSDFL, and LTKPLVSLIQAL was as susceptible to tobramycin as Δ*sagS* mutant biofilms (Fig. 6). In contrast, Δ*sagS* mutants producing SagS variants in which the amino acid residues R116, L141, G152, F167, I173, ITLLSG, L189, L197, or R218 were substituted for with alanine demonstrated an intermediate-susceptibility phenotype (Fig. 6). This was supported by exposure to tobramycin only coinciding with an ~1.8- to 2.2-fold reduction in the viability of the respective Δ*sagS*/pMJT-*sagS*_*mut*_ biofilms. Our findings suggest that out of 44 SagS variants, 27 SagS variants either failed to or only partly restored the susceptibility phenotype of Δ*sagS* mutant biofilms to wild-type levels (Table 1).

It is of interest to note that among the 27 SagS variants affecting biofilm susceptibility, only 4 SagS variants (in which the amino acid residues G132, L156, L189, and LTKPLVSLIQAL were substituted for with alanine) failed to also restore attachment by Δ*sagS* mutant biofilms to wild-type levels (Table 1, Fig. 3, and Fig. 6). The respective variants were excluded from subsequent analysis.

### Substitution for D105, R116, and L126 with alanine eliminates the recalcitrance of Δ*sagS* biofilms to killing by tobramycin.

SagS not only contributes to the resistance of biofilm cells to antimicrobial agents but also to the recalcitrance of biofilm cells to killing by bactericidal antibiotics, with inactivation of *sagS* correlating with complete killing of biofilm cells following 24 h of exposure to tobramycin and norfloxacin at concentrations exceeding 100 µg/ml, in contrast to survival of wild-type cells following the same treatment (30, 36, 37). To determine whether select HmsP domain amino acid residues contribute to the recalcitrance of P. aeruginosa biofilm cells to antimicrobial agents, we made use of biofilm minimum bactericidal concentration (MBC) assays. Specifically, we selected SagS_mut_ variants SagS_D105A, SagS_R116A, SagS_L126A, and SagS_L141A, which were found to either partly or fully restore attachment by Δ*sagS* mutant strains to wild-type levels but fail to restore the susceptibility phenotype of Δ*sagS* mutant biofilms to wild-type levels (Fig. 2, Fig. 3A, Fig. 5, and Table 1). In addition, Δ*sagS* mutant strains expressing *sagS*_F131A and *sagS*_L154A were chosen as they failed to restore attachment to wild-type levels, but rendered Δ*sagS* mutant biofilms resistant to tobramycin (Fig. 2, Fig. 3A, Fig. 5, and Table 1). All biofilms were exposed to increasing concentrations of tobramycin (0, 75, 150, and 300 µg/ml), and the number of surviving cells was determined following 24 h of exposure.

**FIG 7:**
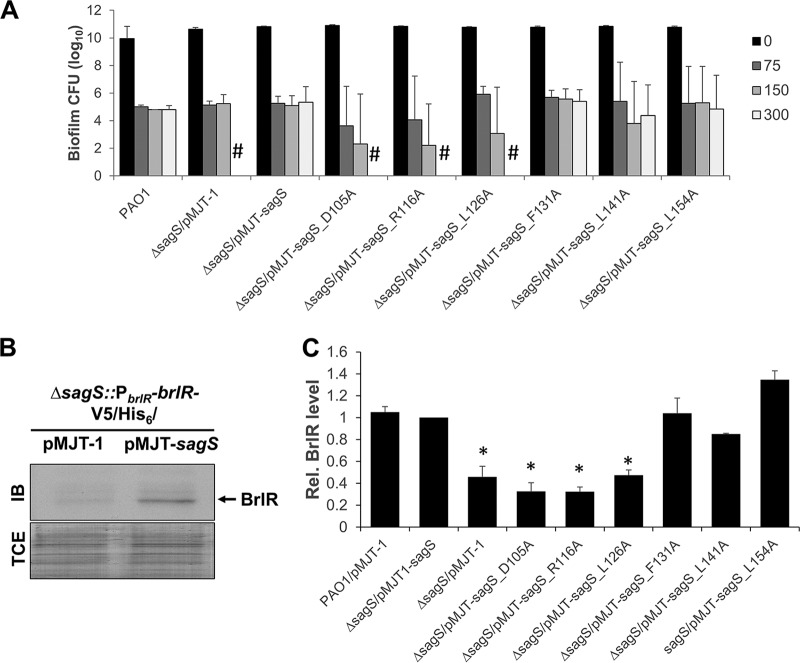
SagS variants contributing to decreased susceptibility of biofilms to tobramycin also contribute to tolerance to tobramycin and BrlR abundance. (A) Biofilm MBC assays. The P. aeruginosa PAO1 wild type or Δ*sagS* and Δ*sagS* mutants expressing *sagS* or select *sagS*_*mut*_ variants were grown as biofilms for 3 days and subsequently treated for 24 h with increasing concentrations of tobramycin (75 to 300 µg/ml) under continuous flow conditions before recovering and enumerating surviving cells. Viability was determined by CFU counts (biofilm CFU obtained from biofilm tube reactors having an inner surface area of 25 cm^2^). #, no viable bacteria were detected. Experiments were carried out in triplicate. Error bars denote standard deviation. (B) Detection of BrlR by immunoblot analysis. Total cell extracts (TCEs) obtained from Δ*sagS*/pMJT*-sagS* and Δ*sagS*/pMJT-1 mutant biofilms (3 days old) expressing a chromosomally located V5/His_6_-tagged BrlR under the control of its own promoter (P_*brlR*_*-brlR-*V5/His_6_) were probed for the presence of BrlR by immunoblot (IB) analysis using anti-V5 antibodies. A total of 15 µg total cell extract was loaded. The corresponding SDS-PAGE gel image obtained posttransfer demonstrates equal loading. Representative images are shown. (C) Relative BrlR level present in biofilms by the PAO1 wild type or Δ*sagS* and Δ*sagS* mutants expressing *sagS* or select *sagS*_*mut*_ variants based on relative intensity of protein bands detectable following probing for BrlR with anti-V5 antibodies and subsequent analysis using ImageJ (56). Experiments were carried out in triplicate. Error bars denote standard deviation. *, significantly different from the values for the Δ*sagS*/pMJT*-sagS* mutant (*P* ≤ 0.01), as determined by ANOVA and Prism5.

In agreement with previous reports (30), *ΔsagS* biofilm cells were eradicated following 24 h of exposure to 150 µg/ml tobramycin, while expression of *sagS* restored the recalcitrance of *ΔsagS* biofilm cells to wild-type levels (Fig. 7A). Despite SagS_F131A being produced at reduced levels (Fig. 4A), expression of *sagS*_F131A restored the recalcitrance of *ΔsagS* biofilms to wild-type levels (Fig. 7A). Likewise, expression of *sagS*_L154A and *sagS*_L141A restored the recalcitrance of *ΔsagS* biofilms to wild-type levels (Fig. 7A), indicating that the three HmsP domain residues play no role in SagS promoting the switch by P. aeruginosa biofilms to a drug tolerance state. In contrast, overexpression of *sagS_*D105A, *sagS*_R116A, and *sagS*_L126A in *ΔsagS* coincided with complete killing of biofilm cells following 24 h of exposure to tobramycin at concentrations exceeding 100 µg/ml (Fig. 7A). This was supported by no viable cells being recovered following 24 h of exposure to tobramycin at concentrations as high as 300 µg/ml (Fig. 7A). The finding suggested a likely role of amino acid residues D105, R116, and L126 in biofilm drug tolerance.

### Expression of *sagS* variants harboring substitutions in D105, R116, and L126 results in reduced BrlR abundance in biofilms.

Drug tolerance by P. aeruginosa biofilms has previously been linked to expression of *brlR* encoding the biofilm resistance regulator BrlR, with inactivation of *sagS* coinciding with reduced *brlR* transcript levels and lack of detectable levels or BrlR (7, 30, 36). We therefore reasoned that substitutions in the SagS-HmsP domain that impair the biofilm tolerance switch function of SagS coincide with reduced BrlR protein abundance, in a manner comparable to that noted for Δ*sagS* mutants.

In agreement with previous findings, *sagS* inactivation coincided with little to no detectable BrlR levels in biofilms, while biofilms by the *ΔsagS*/pMJT-*sagS* mutant were characterized by the presence of BrlR (Fig. 7B and C). Immunoblot analysis further demonstrated that multicopy expression of *sagS*_F131A, *sagS*_L154A, and *sagS*_L141A in biofilms by the *ΔsagS* mutant likewise restored BrlR to wild-type levels (Fig. 7C). In contrast, multicopy expression of *sagS_*D105A, *sagS*_R116A, and *sagS*_L126A in biofilms by *ΔsagS* failed to restore BrlR to wild-type levels (Fig. 7C). Our findings suggested that substitutions of amino acid residues negatively affecting attachment and biofilm formation have no effect on BrlR levels, while amino acid residues contributing to biofilm tolerance likely interfere with SagS contributing to the level of BrlR.

### Structural analysis of SagS and mapping of amino acid residues.

**FIG 8:**
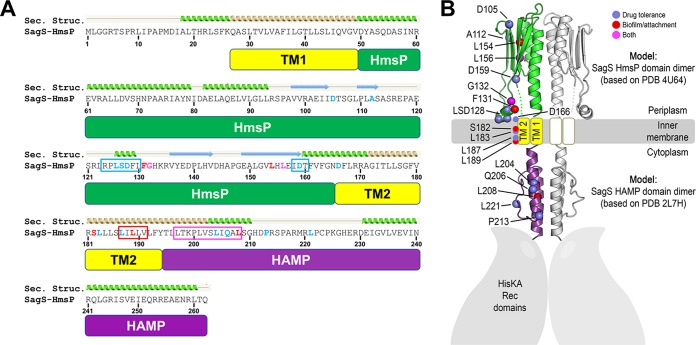
Secondary structure prediction of the HmsP and HAMP domains of SagS and spatial mapping of residues affecting SagS function. (A) Sequence of the sensory HmsP domain of SagS. Based on structural predictions (Sec. Structure) using the PHYRE2 server, the HAMP domain is shown in purple, transmembrane helices in yellow, and the sensory domain in green. Mutations affecting biofilm development (red), biofilm drug tolerance (blue), or both (magenta) are indicated in the alignment. Following the same color code, boxed regions highlight sequence motifs that were targeted for multialanine substitutions. (B) Domain modeling and spatial mutation mapping. Structural predictions were initially carried out using the PHYRE2 server. Homology models of domain dimers were calculated using Rosetta-based homology modeling. The HAMP domain is shown in purple, transmembrane helices in yellow, and the sensory domain in green. Amino acid residues (colored spheres marking C-α positions) that affect SagS function were mapped onto the three-dimensional models. Substitution of amino acids affecting attachment and biofilm formation are indicated in red, while substitutions of residues affecting susceptibility and drug tolerance are shown in blue. Amino acid residues affecting both are shown in magenta.

In summary, of the 44 SagS variants, a total of 36 variants failed to restore attachment and/or resistance to tobramycin to wild-type levels, with 32 SagS variants failing to restore only one of the two phenotypes tested (Table 1). Analysis of the amino acid sequence of HmsP revealed no clustering of the two sets of amino acids (Fig. 8A). To determine whether residues affecting biofilm formation (or biofilm drug tolerance) may be spatially arranged, we carried out structural prediction analysis of SagS, focusing on its periplasmic HmsP and cytoplasmic HAMP domains. PHYRE2 supported the notion that the domain following the second transmembrane domain of SagS can adopt a characteristic HAMP fold (top-ranked model: 24% sequence identity to PDB 2L7H with 99.3% model confidence) (Fig. 8A) (44). A HAMP domain is commonly found in the cytoplasmic portion of a transmembrane protein and is hypothesized to play a role in relaying signals from the periplasmic to the cytoplasmic domains of a protein (45).

The analysis also predicted a periplasmic sensory subdomain similar to the periplasmic domain of the c-di-GMP sensor LapD (number 2-ranked model: 11% sequence identity to PDB 4U65 with 97.9% model confidence) (46). Similar to SagS, the periplasmic domain of LapD is flanked by two transmembrane helices (Fig. 2B and 8A) (47). Although the overall sequence identity is low between the periplasmic domains of the two proteins, the predicted secondary structure motif of SagS aligns with corresponding elements in the LapD crystal structure, correlating with a high confidence score for the structural model. LapD’s periplasmic domain belongs to the family of Cache domains that comprise the largest superfamily of extracellular sensors in bacteria according to a recent bioinformatics study and associated census (48). Together, this analysis suggests that the HmsP domain of SagS may adopt a Cache domain fold, consistent with its presumed role as a sensory module.

For the mapping of mutations on the predicted structures of the SagS domains, we created molecular models by using the Rosetta computational modeling package. The resulting models correlated well with those generated by the PHYRE2 server. Figure 8B shows a composite SagS dimer containing atomistic models for the periplasmic HmsP sensing (green) and HAMP (purple) domains. Transmembrane segments (yellow) are illustrated as well. We next mapped the two sets of amino acid residues onto the predicted structures of SagS domains, with amino acids affecting attachment and biofilm formation in red and those affecting susceptibility and drug tolerance in blue (Fig. 8B). Amino acid residues affecting both are shown in magenta (Fig. 8B). Notably, none of the residues of interest were found along the vertical helix of the periplasmic sensory domain, which in LapD and similar proteins serves as the domain’s main dimerization motif. Instead, mutations targeted to a predicted membrane-proximal helix (i.e., L126, S127, and D128) appear to affect drug tolerance specifically. Other mutations with a similar phenotype (D105, A112, and D159) locate to the beta-sheet region of the fold, which is a site of ligand binding in other sensing domains (49). The HAMP domain also appears to be particularly sensitive with regard to perturbations leading to altered drug tolerance, with several mutations likely impacting the switching mechanism of this conformational relay module (Fig. 8B). Mutations affecting biofilm development are scarcer and are distinct from those affecting drug sensitivity, yet they locate to similar regions of the protein, namely, the beta-sheet of the HmsP domain and the HAMP domain. Interestingly, in this model, the two mutations that affect both phenotypes similarly (i.e., G132 and L156) are flanked by a residue that is crucial for biofilm regulation on one side and a residue important for drug sensitivity on the other site. The second predicted transmembrane helix harbors residues important for biofilm formation and drug tolerance, but yet again, the mutations confer distinct phenotypes, suggesting that different conformations of SagS are associated with its signaling outcome. The presence of multialanine substitutions spanning about 3 helical turns of the first HAMP domain helix (i.e., the LTKPLVSLIQAL motif) results in a mixed phenotype affecting both drug tolerance and biofilm formation, further confirming the importance of this domain in the global regulation of SagS.

## DISCUSSION

We initiated this study to determine whether there are amino acid residues present in the HmsP domain that specifically contribute to the switch function of SagS. This was accomplished by alanine substitutions for conserved amino acid residues or extended sequences within the HmsP domain. Of the 44 SagS variants tested, a total of 36 variants failed to restore attachment and/or resistance to tobramycin to wild-type levels. More importantly, of the 36 variants, 32 SagS variants failed to restore only one of the two phenotypes tested (Table 1). The findings suggested that by substituting amino acids, SagS function can be blocked with respect to the sensory function(s) of HmsP, which is apparent by attached cells being unable to (i) develop mature biofilms and/or (ii) by preventing transition to an antimicrobial-resistant state.

Our finding furthermore suggested the presence of a set of amino acid residues in the SagS-HmsP domain that contribute to attachment and subsequent biofilm formation, but not biofilm drug tolerance, while a second set of amino acids contribute to biofilm drug tolerance but not attachment and subsequent biofilm formation. For instance, SagS variants harboring alanine substitutions in residues F131 and L154 were found to be unable to restore attachment, biofilm formation, and BfiS phosphorylation by *ΔsagS* mutant strains to wild-type levels (Table 2). In contrast, SagS variants harboring alanine substitutions in residues D105, R116, and L126 were unable to restore susceptibility to tobramycin, drug tolerance, and BrlR levels by *ΔsagS* mutant strains to wild-type levels (Table 2). Our findings thus strongly supported the notion of the periplasmic sensory domain HmsP contributing to SagS function, with the HmsP domain likely being a control point in the regulation of biofilm development and biofilm cells transitioning from a susceptible to an antimicrobial-resistant state.

**TABLE 2:** Overview of biofilm-related phenotypes of select SagS variants

Substitution	Restoration of Δ*sagS* biofilm-related phenotypes to wild-type levels^a^					
	Attachment	Biofilm architecture	BfiS phosphorylation	Susceptibility to tobramycin	Tolerance to tobramycin	BrlR levels
D105	Yes	Yes	Yes	No	No	No
L126	Yes	Yes	Yes	No	No	No
F131	No	No	No	Yes	Yes	Yes
L154	No	No	No	Yes	Yes	Yes
L141	Yes	Yes	Yes	Intermediate	Yes	Yes
R116	Intermediate	Yes	Yes	Intermediate	No	No

a The wild type and Δ*sagS* mutant strain harboring the empty vector pMJT-1 or overexpressing *sagS* were used as controls. Data correspond to results shown in Fig. 3 to 7. “Yes” indicates restoration of Δ*sagS* biofilm-related phenotypes to wild-type levels, “No” indicates no restoration to wild-type levels, and “Intermediate” indicates values or phenotypes were in between those obtained for the PAO1 wild type or Δ*sagS*/pMJT-*sagS* and *ΔsagS* mutants.

The location of amino acids affecting SagS phenotypes provided additional clues for how the sensory protein SagS may relay cues from the periplasmic HmsP domain to the intracellular compartment. The finding that distinct residues located in the predicted HmsP, transmembrane, and HAMP domains contribute to biofilm formation or antibiotic tolerance likely suggests that SagS transduces a signal to the HAMP domain via distinct conformational changes. Only a few residues affect both phenotypes equally when mutated. Based on the model, these positions are flanked by residues with single, yet distinct mutation phenotypes, which may suggest that mutating the connecting regions (i.e., positions marked in magenta in Fig. 8B) impacts the communication and therefore the switching within SagS. Such a model is supported by the observation that a more severe multialanine substitution in the HAMP domain also affects both phenotypes similarly, whereas single mutations affect one or the other phenotype (Fig. 8B). Taken together, our structural modeling suggests that SagS adopts distinct conformations that are associated with different signaling states and physiological outcomes.

Mutations affecting drug tolerance that locate to the periplasmic RPLSDFL motif (residues 124 to 130 of SagS) mark a peculiar structural feature in the HmsP domain homology model (Fig. 8B). The motif adopts a short helical conformation that was also observed in the crystal structure of LapD’s periplasmic domain (46) and that, based on our models, is membrane proximal. In a recent structural study on the sensor histidine kinase NarQ, Gushchin and colleagues (50) noted a small loop of the sensor domain corresponding in position to the aforementioned motif in SagS (and LapD). In NarQ, this loop is anchored into the membrane via hydrophobic residues (Trp and Tyr). It is thus likely that hydrophobic residues in the SagS PLSDFLF motif (including residues L126 and F131) fulfill a similar structural role, and based on our mutational analysis, also function in receptor switching.

The classification of the HmsP domain as a Cache-like sensing domain is based solely on fold predictions, which suggested homology to the periplasmic domain of P. fluorescens or Legionella pneumophila LapD (46). LapD contributes to the transition from the reversible to the irreversible attachment stage, and thus, biofilm formation, by controlling the outer membrane association of the LapA adhesin (51). The two proteins differ, however, in their cytoplasmic C-terminal domains, with SagS harboring a histidine kinase (HisKA) and receiver (Rec) domains, while LapD harbors GGDEF and EAL domains (47, 52). However, similar to SagS, LapD is predicted to be localized in the cytoplasmic membrane via two transmembrane helices that flank the protein’s periplasmic domain. Both, SagS and LapD contain a juxtamembrane HAMP domain, which may suggest a conserved switching mechanism. Cache domains have also been predicted to comprise the most prevalent fold for extracellular sensor function (48), further supporting our modeling results. Nevertheless, experimental structure determination will be required to confirm this annotation or provide a more accurate classification that may also yield further insight into the protein switching mechanism and potential ligands controlling biofilm development and biofilm cells transitioning from a susceptible to an antimicrobial-resistant state.

Overall, our findings strongly support the notion of the periplasmic sensory HmsP domain contributing to SagS function, with the HmsP domain likely being a control point in the regulation of biofilm development and biofilm cells transitioning from a susceptible to an antimicrobial-resistant state. Moreover, our findings suggest SagS to be a potential target for the treatment of biofilm-related P. aeruginosa infections, with treatment based on interfering with or blocking HmsP-based signaling being anticipated to result in biofilm cells being unable to transition to a resistant state.

## MATERIALS AND METHODS

### Bacterial strains, plasmids, and culture conditions.

All bacterial strains and plasmids used in this study are listed in Table S1 in the supplemental material. Bacterial overnight cultures were grown at 37°C in Lennox broth (LB) medium (BD Biosciences) in flasks with continuous shaking at 220 rpm. Biofilms were grown at room temperature in 20-fold-diluted LB. Plasmids were maintained by supplementing LB with ampicillin (100 µg/ml) for growth of Escherichia coli or carbenicillin (250 µg/ml) and gentamicin (50 µg/ml) for growth of Pseudomonas aeruginosa.

10.1128/mSphere.00192-18.2 TABLE S1  Bacterial strains and plasmids. Download TABLE S1, DOCX file, 0.1 MB. Copyright © 2018 Dingemans et al. 2018 Dingemans et al. This content is distributed under the terms of the Creative Commons Attribution 4.0 International license.

### Strain construction.

The rationale behind this study was to identify amino acids located in the periplasmic sensory domain (HmsP) of SagS that were involved in biofilm formation and/or tolerance. To do this, we first aligned the HmsP domain of Yersinia pestis to the HmsP domain of BifA and SagS in Pseudomonas aeruginosa to identify conserved residues located between the predicted transmembrane domains and hence potentially exposed to the periplasm (Fig. 2B and 8A). Besides 38 conserved residues, six regions were identified that contained two or more conserved residues. Conserved residues were substituted for with alanine (or serine if the conserved residue was an alanine) by means of the GeneArt site-directed mutagenesis kit (Invitrogen) and the Q5 site-directed mutagenesis kit (New England Biolabs) according to the manufacturer’s protocol. Furthermore, alanine cassette mutants were constructed in regions that contained two or more conserved residues. The pET vector harboring the wild-type *sagS* gene was used as a template for construction of the mutants. Site-directed mutated variants of *sagS* were subsequently subcloned into the pMJT-1 vector using the oligonucleotides listed in Table S2 in the supplemental material and the restriction enzymes NheI-HF and SacI-HF (New England Biolabs). Finally, the P. aeruginosa Δ*sagS* strain was complemented with wild-type *sagS* or each of the mutated versions of *sagS* in the pMJT1 vector.

10.1128/mSphere.00192-18.3 TABLE S2  Oligonucleotides used. Download TABLE S2, DOCX file, 0.1 MB. Copyright © 2018 Dingemans et al. 2018 Dingemans et al. This content is distributed under the terms of the Creative Commons Attribution 4.0 International license.

### Attachment assay.

Overnight cultures of each strain/mutant were diluted 100-fold in LB medium containing 250 µg/ml carbenicillin to an optical density at 600 nm (OD_600_) of 0.025. Then, 96-well plates were inoculated with 200 µl of the diluted culture and incubated for 24 h at 37°C with continuous shaking at 220 rpm. Next, 50 µl of crystal violet was added, followed by incubation for 15 min at 37°C with continuous shaking at 220 rpm. Plates were washed three times with water and allowed to dry prior to the addition of 200 µl of ethanol to each well and subsequent incubation for 15 min at 37°C with continuous shaking at 220 rpm. Finally, the OD_575_ was determined. Blank values (LB medium alone) were subtracted, and data were normalized to the values obtained for P. aeruginosa Δ*sagS* overexpressing wild-type *sagS*.

### Biofilm growth.

For biofilm antibiotic susceptibility, biofilm MBC assays, protein extraction, and RNA extraction, biofilms were grown in a once-through continuous flow reactor system with size 13 (inner surface area, 25 cm^2^) Masterflex silicone tubing (Cole Parmer) for 3 days at a flow rate of 0.1 ml/min, as previously described using 20-fold-diluted LB medium (3–5). To maintain plasmids, carbenicillin at 10 µg/ml was added to the growth medium. For visualization of the biofilm architecture, biofilms were grown in 24-well plates in 5-fold-diluted LB medium containing 10 µg/ml carbenicillin, with the growth medium being exchanged every 12 h. Confocal laser scanning microscopy (CLSM) images were acquired using a Leica TCS SP5 confocal microscope (Leica Microsystems, Inc., Wetzlar, Germany) and the LIVE/DEAD BacLight bacterial viability kit (Life Technologies, Inc.). Quantitative analysis of the confocal laser scanning microscope images of 24-well plate-grown biofilms was performed using COMSTAT (7). For all biofilm growth conditions, wild-type strain PAO1 and the *ΔsagS* mutant strain harboring empty plasmids were used as vector controls.

### Phosphoprotein enrichment and detection.

To study phosphorylation of BfiS by SagS under biofilm conditions, a pJN-*bfiS*-V5/His_6_ construct was introduced in each complemented Δ*sagS* mutant. Biofilms were grown in 20-fold-diluted LB containing 10 µg/ml carbenicllin, 2 µg/ml gentamicin, and 0.1% arabinose for 3 days under flowing conditions (0.2 ml/min), harvested by extrusion of the cell paste, and resuspended in 500 µl of TE buffer (10 mM Tris-HCl [pH 8.0], 1 mM EDTA) plus 0.3 µg/ml phenylmethylsulfonyl fluoride (PMSF), followed by lysis via sonication. Phosphorylated proteins were enriched via metal oxide affinity chromatography (MOAC) essentially as described by Wolschin and colleagues (53). MOAC has been demonstrated by Krüger et al. to result in up to 20-fold enrichment of phosphoproteins and to approach 100% specificity (54). Briefly, 750 µg of total cell extract (TCE) was incubated with MOAC incubation buffer, consisting of 30 mM MES (morpholineethanesulfonic acid), 0.2 M potassium glutamate, 0.2 M sodium aspartate, 0.25% CHAPS {3-[(3-cholamidopropyl)-dimethylammonio]-1-propanesulfonate, and 8 M urea in a final volume of 1.5 ml, and subsequently incubated for 30 min at 4°C in the presence of 80 mg of aluminum hydroxide. Unbound proteins were removed via six 1-min washes with 1.5 ml of incubation buffer at 16,000 × *g* and 4°C. Next, phosphoproteins were eluted from the slurry using 100 mM potassium pyrophosphate and 8 M urea, desalted by methanol-chloroform precipitation, and subsequently vacuum dried. Finally, samples were analyzed by SDS-PAGE, followed by the detection of BfiS-V5/His_6_ by immunoblotting with anti-V5 antibodies as described above. Aliquots obtained prior to MOAC were used as loading controls.

### Biofilm antibiotic susceptibility testing.

Susceptibility of biofilm cells to antibiotic treatment was determined by growing biofilms for 2 days using a continuous flow reactor. Under the conditions tested, a total of 1.1 × 10^10^ CFU/biofilm were obtained on average following 2 days of biofilm growth. Biofilm cells in the continuous flow reactor were then exposed to tobramycin (150 µg/ml) for 1 h under flowing conditions. Upon completion of the treatment, the cells were harvested and homogenized to disrupt any aggregates and cell clusters. The homogenized cells were serially diluted, and up to 7 dilutions were plated onto LB agar. Following overnight incubation at 37°C, CFU were determined. Viability was determined via CFU counts and susceptibility is expressed as log_10_ reduction.

### Biofilm MBC assay.

The minimum bactericidal concentration (MBC) is the concentration required to prevent the growth of bacterial cells in antibiotic-free medium after exposure to antibiotic (8). The biofilm MBC is defined as the concentration at which no further increase in log reduction is observed (9–11). To determine biofilm MBC, biofilm cells were grown for 3 days, after which the medium was switched to one containing tobramycin at concentrations ranging from 75 to 300 µg/ml. The chosen concentration range has previously been shown to demonstrate recalcitrance to killing by wild-type biofilm cells, while complete eradication was achieved for Δ*brlR* mutant biofilm cells (6). The cells were treated for 24 h and subsequently harvested, homogenized, and plated to measure viability.

### Structure prediction of the HmsP and HAMP domains of SagS.

The amino acid sequences of the isolated HmsP and HAMP domains of SagS were submitted to the PHYRE2 Protein Fold Recognition Server (57), yielding a prediction model of the structure of the HmsP domain. Dimer homology models were calculated using the Rosetta modeling package (55) and PDB codes 4U64 (46) and 2L7H (44) as the templates. Default parameters were used during PHYRE2- and Rosetta-based modeling. The resulting models were visualized using Pymol.

### Statistical analysis.

To determine differences between mutants, one-way analysis of variance (ANOVA) was performed, followed by a Tukey’s test *a posteriori* to compare the means of all treatment groups. All statistical analyses were performed using the Prism5 software (Graph Pad, La Jolla, CA).

### Data availability.

Strains, plasmids, and other data will be provided upon request.
