# Supplementary figures and images for: The Yin and Yang of SagS: Distinct Residues in the HmsP Domain of SagS Independently Regulate Biofilm Formation and Biofilm Drug Tolerance
Source: mSphere. 2018 May 30;3(3):e00192-18. doi: 10.1128/mSphere.00192-18 (PMC5976881; doi:10.1128/mSphere.00192-18)

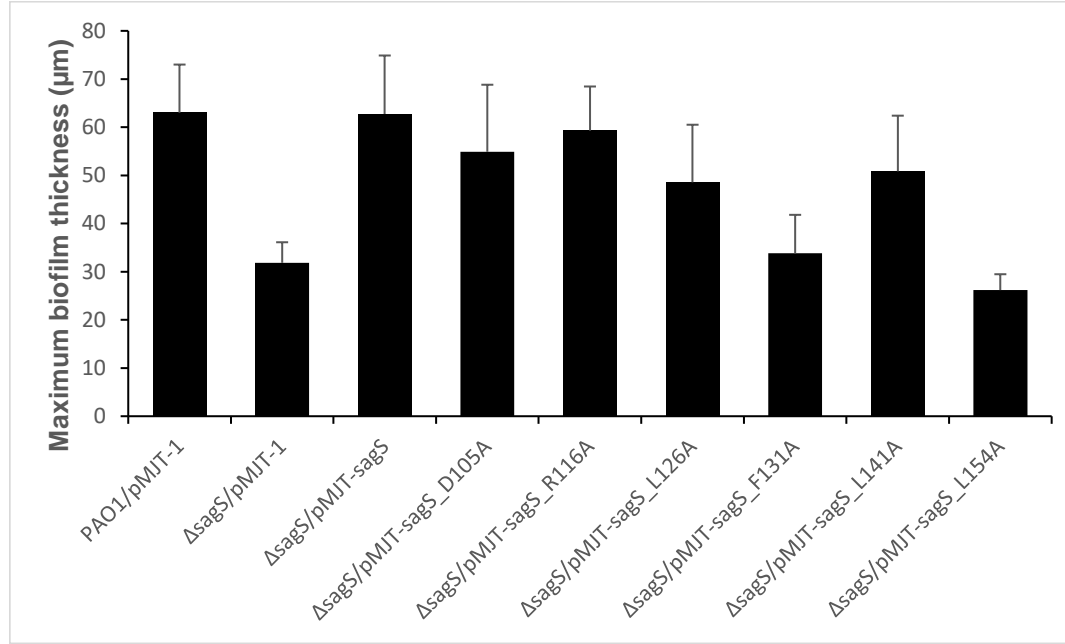

**Figure S1.**

Supplement: FIG S1 [file sph003182558sf1.pdf]
