# Supplementary material for: The Yin and Yang of SagS: Distinct Residues in the HmsP Domain of SagS Independently Regulate Biofilm Formation and Biofilm Drug Tolerance
Source: mSphere. 2018 May 30;3(3):e00192-18. doi: 10.1128/mSphere.00192-18 (PMC5976881; doi:10.1128/mSphere.00192-18)
Supplement: TABLE S1 [file sph003182558st1.docx]

**Table S1**

| Strains/Plasmids | Relevant genotype or description | Source |
| --- | --- | --- |
| Strains |  |  |
| *Escherichia coli* | | |
| DH5α | *F- φ80lacZ∆M15 ∆(lacZYA-argF)U169 recA1 endA1 hsdR17(rk-, mk+) phoA supE44 thi-1 gyrA96 relA1 tonA* | Invitrogen Corp |
| *P. aeruginosa* | | |
| PAO1 | Wild type | B.H. Holloway |
| *ΔsagS* | PAO1, ΔsagS (PA2824) | ([1](#_ENREF_1)) |
| Plasmids | | |
| pCR2.1-TOPO® | TA cloning vector; Km^R^; Amp^R^ | Invitrogen Corp |
| pET101D | Vector for directional cloning and high level V5/6XHis fusion protein expression, Amp^R^ | Invitrogen Corp |
| pMJT-1 | *araC*-P_BAD_ cassette of pJN105 cloned into pUCP18, Amp^R^ (Carb^R^) | ([2](#_ENREF_2)) |
| pMJT-*sagS* | Arabinose-inducible expression of *sagS* in PAO1; Amp^R^ (Carb^R^) | ([3](#_ENREF_3)) |
| pMJT-*sagS_mut_* | Arabinose-inducible expression of *sagS_mut_* in PAO1; Amp^R^ (Carb^R^) | This study |

**REFERENCES**

1. **Petrova OE, Sauer K.** 2011. SagS contributes to the motile-sessile switch and acts in concert with BfiSR to enable *Pseudomonas aeruginosa* biofilm formation J. Bacteriol. **193:**6614-6628.

2. **Kaneko Y, Thoendel M, Olakanmi O, Britigan BE, Singh PK.** 2007. The transition metal gallium disrupts *Pseudomonas aeruginosa* iron metabolism and has antimicrobial and antibiofilm activity. J. Clin. Invest. **117:**877-888.

3. **Petrova OE, Gupta K, Liao J, Goodwine JS, Sauer K.** 2017. Divide and conquer: the *Pseudomonas aeruginosa* two-component hybrid SagS enables biofilm formation and recalcitrance of biofilm cells to antimicrobial agents via distinct regulatory circuits. Environmental Microbiology **19:**2005-2024.
