# Supplementary material for: The Yin and Yang of SagS: Distinct Residues in the HmsP Domain of SagS Independently Regulate Biofilm Formation and Biofilm Drug Tolerance
Source: mSphere. 2018 May 30;3(3):e00192-18. doi: 10.1128/mSphere.00192-18 (PMC5976881; doi:10.1128/mSphere.00192-18)
Supplement: TABLE S2 [file sph003182558st2.docx]

**Table S2**

| Oligonucleotide | Sequence |
| --- | --- |
| Single amino acid residue substitutions | |
| sagS-I103A_for | CCGCCGTGGTCCGCGCGGAAGCCATCGACACCTCCGGCCTGCC |
| sagS-I103A_rev | GGCAGGCCGGAGGTGTCGATGGCTTCCGCGCGGACCACGGCGG |
| sagS-D105A_for | GAAATCATCGCCACCTCCGGCCTGCCGC |
| sagS-D105A rev | CGCGCGGACCACGGCGGG |
| sagS-A112S_for | ACACCTCCGGCCTGCCGCTGTCCAGCGCCTCGCGCGAACCCGC |
| sagS-A112S_rev | GCGGGTTCGCGCGAGGCGCTGGACAGCGGCAGGCCGGAGGTGT |
| sagS-S113A_for | GCCGCTGGCCGCCGCCTCGCGC |
| sagS-S113A_rev | AGGCCGGAGGTGTCGATGATTTC |
| sagS-R116A_for | CAGCGCCTCGGCCGAACCCGCCGAAAG |
| sagS-R116A_rev | GCCAGCGGCAGGCCGGAG |
| sagS-R124A for | GAACCCGCCGAAAGCCGCCTGGCGCCGCTCAGCGACTTCCTCTTC |
| sagS-R124A_rev | GAAGAGGAAGTCGCTGAGCGGCGCCAGGCGGCTTTCGGCGGGTTC |
| sagS-L126A_for | CGAAAGCCGCCTGCGGCCGGCCAGCGACTTCCTCTTCGGCC |
| sagS-L126A_rev | GGCCGAAGAGGAAGTCGCTGGCCGGCCGCAGGCGGCTTTCG |
| sagS-S127A_for | GAAAGCCGCCTGCGGCCGCTCGCCGACTTCCTCTTCGGCCACAAG |
| sagS-S127A_rev | CTTGTGGCCGAAGAGGAAGTCGGCGAGCGGCCGCAGGCGGCTTTC |
| sagS-D128A_for | CCGCTCAGCGCCTTCCTCTTCGG |
| sagS-D128A_rev | CCGCAGGCGGCTTTCGGC |
| sagS-L130A_for | CAGCGACTTCGCCTTCGGCCACAAGCGGGTCTAC |
| sagS-L130A_rev | AGCGGCCGCAGGCGGCTT |
| sagS-F131A_for | CGGCCGCTCAGCGACTTCCTCGCCGGCCACAAGCGGGTCTACGAG |
| sagS-F131A_rev | CTCGTAGACCCGCTTGTGGCCGGCGAGGAAGTCGCTGAGCGGCCG |
| sagS-G132A_for | TTCCTCTTCGCCCACAAGCGGGTCTAC |
| sagS-G132A_rev | GTCGCTGAGCGGCCGCAG |
| sagS-K134A_for | CTTCGGCCACGCGCGGGTCTACG |
| sagS-K134A_rev | AGGAAGTCGCTGAGCGGC |
| sagS-L141A_for | CGAGGACCCGGCGCATGTCGACC |
| sagS-L141A_rev | TAGACCCGCTTGTGGCCG |
| sagS-G152A_for | GAGGCCCTCGCCGTACTGCACC |
| sagS-G152A_rev | GCCCGGCGCATGGTCGAC |
| sagS-L154A_for | CGGGCGAGGCCCTCGGCGTAGCGCACCTGGAAATCGACACCTTC |
| sagS-L154A_rev | GAAGGTGTCGATTTCCAGGTGCGCTACGCCGAGGGCCTCGCCCG |
| sagS-L156A_for | GAGGCCCTCGGCGTACTGCACGCGGAAATCGACACCTTCGTGTTC |
| sagS-L156A_rev | GAACACGAAGGTGTCGATTTCCGCGTGCAGTACGCCGAGGGCCTC |
| sagS-D159A_for | GGCGTACTGCACCTGGAAATCGCCACCTTCGTGTTCGGCAACGAC |
| sagS-D159A_rev | GTCGTTGCCGAACACGAAGGTGGCGATTTCCAGGTGCAGTACGCC |
| sagS-D166A_for | GACACCTTCGTGTTCGGCAACGCCTTCCTCCGTCGCGCCGGCATC |
| sagS-D166A_rev | GATGCCGGCGCGACGGAGGAAGGCGTTGCCGAACACGAAGGTGTC |
| sagS-F167A_for | CGGCAACGACGCCCTCCGTCGC |
| sagS-F167A_rev | AACACGAAGGTGTCGATTTCC |
| sagS-I173A_for | GACTTCCTCCGTCGCGCCGGCGCCACCCTGCTCTCCGGCTTCGTG |
| sagS-I173A_rev | CACGAAGCCGGAGAGCAGGGTGGCGCCGGCGCGACGGAGGAAGTC |
| sagS-S177A_for | CGCGCCGGCATCACCCTGCTCGCCGGCTTCGTGCGCAGCCTGCTG |
| sagS-S177A_rev | CAGCAGGCTGCGCACGAAGCCGGCGAGCAGGGTGATGCCGGCGCG |
| sagS-V180A_for | ATCACCCTGCTCTCCGGCTTCGCGCGCAGCCTGCTGCTGTCGCTG |
| sagS-V180A_rev | CAGCGACAGCAGCAGGCTGCGCGCGAAGCCGGAGAGCAGGGTGAT |
| sagS-S182A_for | CTGCTCTCCGGCTTCGTGCGCGCCCTGCTGCTGTCGCTGATCCTG |
| sagS-S182A_rev | CAGGATCAGCGACAGCAGCAGGGCGCGCACGAAGCCGGAGAGCAG |
| sagS-S183A_for | CTCTCCGGCTTCGTGCGCAGCGCGCTGCTGTCGCTGATCCTGCTG |
| sagS-S183A_rev | CAGCAGGATCAGCGACAGCAGCGCGCTGCGCACGAAGCCGGAGAG |
| sagS-L185A_for | GGCTTCGTGCGCAGCCTGCTGGCGTCGCTGATCCTGCTGGTGCTC |
| sagS-L185A_rev | GAGCACCAGCAGGATCAGCGACGCCAGCAGGCTGCGCACGAAGCC |
| sagS-L187A_for | GCTGCTGTCGGCGATCCTGCTGGTGCTC |
| sagS-L187A_rev | AGGCTGCGCACGAAGCCG |
| sagS-L189A_for | GTCGCTGATCGCGCTGGTGCTCTTCTATACCCTG |
| sagS-L189A_rev | AGCAGCAGGCTGCGCACG |
| sagS-V191A_for | ATCCTGCTGGCGCTCTTCTATACCC |
| sagS-V191A_rev | CAGCGACAGCAGCAGGCT |
| sagS-L197A_for | CTATACCCTGGCGACCAAGCCC |
| sagS-L197A_rev | AAGAGCACCAGCAGGATC |
| sagS-T198A_for | TACCCTGCTGGCCAAGCCCCT |
| sagS-T198A_rev | TAGAAGAGCACCAGCAGGATCAG |
| sagS-L204A_for | CCTGGTCAGCGCGATCCAGGCCC |
| sagS-L204A_rev | GGCTTGGTCAGCAGGGTA |
| sagS-Q206A_for | CAGCCTGATCGCGGCCCTCAGCGG |
| sagS-Q206A_rev | ACCAGGGGCTTGGTCAGC |
| sagS-L208A_for | GATCCAGGCCGCCAGCGGACACG |
| sagS-L208A_rev | AGGCTGACCAGGGGCTTG |
| sagS-D212A_for | AGCGGACACGCTCCACGCTCG |
| sagS-D212A_rev | GAGGGCCTGGATCAGGCTG |
| sagS-P213A_for | CGGACACGATGCACGCTCGCC |
| sagS-P213A_rev | CTGAGGGCCTGGATCAGGC |
| sagS-R218A_for | CTCGCCGGCAGCCATGCGCCTG |
| Sags-R218A_rev | CGTGGATCGTGTCCGCTG |
| sagS-L221A_for | GCTCGCCGGCACGCATGCGCGCGCCCTGCCCCAAGGGCCACGAAC |
| sagS-L221A_rev | GTTCGTGGCCCTTGGGGCAGGGCGCGCGCATGCGTGCCGGCGAGC |
| Cassette substitutions | |
| sagS-LASASR_for | gccgcggcgGAACCCGCCGAAAGCCGC |
| sagS-LASASR_rev | ggcggccgcCGGCAGGCCGGAGGTGTC |
| sagS-RPLSDFL_for | cgccgccgccTTCGGCCACAAGCGGGTC |
| sagS-RPLSDFL_rev | gcggccgccgcCAGGCGGCTTTCGGCGGG |
| sagS-IDT_for | cgccTTCGTGTTCGGCAACGAC |
| sagS-IDT_rev | gcggcTTCCAGGTGCAGTACGCC |
| sagS-ITLLSG_for | gccgccgccTTCGTGCGCAGCCTGCTGC |
| sagS-ITLLSG_rev | cgcggcggcGCCGGCGCGACGGAGGAA |
| sagS-LILLV_for | ggcggcgCTCTTCTATACCCTGCTGACCAAGC |
| sagS-LILLV_rev | gcggccgcCGACAGCAGCAGGCTGCG |
| sagS-LTKPLVSLIQAL for | gccgcggccgcggccgccAGCGGACACGATCCACGC |
| sagS-LTKPLVSLIQAL rev | ggccgcggccgcggccgcCAGGGTATAGAAGAGCACCAGC |
| Cloning | |
| sagS_NheI for | GCGCGCGCgctagcATGCTAGGCGGCAGAACCTCGC |
| sagS_HA_SacI rev | GCGCGCGCgagctcCTAagcgtagtctgggacgtcgtatgggtaGTCGCTCGCGGTGAGCGG |
